# Understanding the physical activity promotion behaviours of podiatrists: a qualitative study

**DOI:** 10.1186/1757-1146-6-S1-O9

**Published:** 2013-05-31

**Authors:** Paul Crisford, Tania Winzenberg, Alison Venn, Verity Cleland

**Affiliations:** 1 Menzies Research Institute Tasmania, Hobart, Australia

## Background

Health professionals are encouraged to play a part in reducing health risks of physical inactivity. Little is known about the factors associated with podiatrists incorporating physical activity promotion into clinical practice.

## Methods

We performed 20 semi-structured interviews with purposefully selected podiatrists to explore their physical activity promotion attitudes, beliefs, knowledge and practice. Transcribed interviews were coded using an iterative thematic approach to identify major themes and salient beliefs.

## Results

Overall podiatrists had a positive attitude to physical activity promotion considering it a normal part of their role. They saw their role as giving information, encouraging activity and making recommendations, however in practice, were less inclined to follow up on recommendations, monitor activity levels or document the process. Their approach was generally opportunistic, informal and un-structured and the content of assessment and promotion dependent upon the presenting patient’s condition. Advice tended to be tailored to the patient’s capabilities and interests. They considered there are opportunities to promote physical activity during regular consultations however were more likely to do so in patients with chronic diseases such as diabetes. Main barriers to physical activity promotion included unreceptive and unmotivated patients as well as a lack of time, skills and resources.

## Conclusion

Physical activity promotion appears feasible in podiatry practice in terms of opportunity and acceptability to practitioners, but there is scope for improvement. Strategies to be employed need to consider the major issues, barriers and opportunities as well as a more structured approach to physical activity promotion by podiatrists.

